# Cholera—Modern Pandemic Disease of Ancient Lineage

**DOI:** 10.3201/eid1711.111109

**Published:** 2011-11

**Authors:** J. Glenn Morris

**Affiliations:** Author affiliation: University of Florida, Gainesville, Florida, USA

**Keywords:** cholera, Vibrio cholerae, bacteria, hyperinfectious, mathematical models, reservoir, synopsis, pandemic, environmental, triggers, human, lineage

## Abstract

Cholera has affected humans for at least a millennium and persists as a major cause of illness and death worldwide, with recent epidemics in Zimbabwe (2008–2009) and Haiti (2010). Clinically, evidence exists of increasing severity of disease linked with emergence of atypical *Vibrio cholerae* organisms that have incorporated genetic material from classical biotype strains into an El Tor biotype background. A key element in transmission may be a recently recognized hyperinfectious phase, which persists for hours after passage in diarrheal feces. We propose a model of transmission in which environmental triggers (such as temperature) lead to increases in *V. cholerae* in environmental reservoirs, with spillover into human populations. However, once the microorganism is introduced into a human population, transmission occurs primary by “fast” transmission from person to person (taking advantage of the hyperinfectious state), without returning to the aquatic environment.

## Abstract 2

Environmental triggers may lead to increases in *Vibrio cholerae* in environmental reservoirs, with spillover into human populations.

Cholera has been an unwanted companion among human civilizations for at least a millennium, with suggestions that it has existed in India “since immemorial times” (*1*). Its impact in Bengal society was sufficient to have resulted in recognition of a goddess of cholera, Oladevi (or Oola Beebee), who required propitiation to protect villages from the disease (*2*). Global pandemic spread of cholera from its ancestral home in Bengal was first documented in 1817 (*1*), the beginning of what has been designated as the first pandemic. In the intervening 2 centuries, cholera has continued to ebb and flow from southern Asia to other parts of the known world, with 6 additional pandemics identified. During the third pandemic, which ravaged London in 1854, John Snow conducted his pioneering epidemiologic studies (and gained fame for removal of a pump handle). We are currently in the throes of the seventh pandemic (caused by *V. cholerae* of the El Tor biotype), which originated almost 50 years ago in the Celebes. In contrast to the earlier 6 pandemics, at no time in these past 50 years has cholera retreated to its southern Asian home. It has instead established endemicity at multiple sites around the globe and continues to trigger major localized epidemics, including the epidemics in Zimbabwe during 2008–2009 (*3*) and Haiti during 2010.

In 2009, the most recent year for which data are available, 221,226 cholera cases were reported to the World Health Organization (WHO) from 45 countries (*4*). This number includes 4,946 deaths, for a case-fatality rate of 2.24%. Although the disease was reported from all continents, 98% of cases reported during 2009 were from Africa, driven in part by large numbers from the latter part of the 2008–2009 Zimbabwe epidemic. However, these numbers should be interpreted with caution because of well-recognized problems with underreporting in the WHO system, particularly because cholera is no longer a notifiable disease and countries can choose whether to report cases. In 2 examples, no cholera cases were included in the annual WHO cholera summary report for 2009 (*4*) from India or Bangladesh, despite anecdotal evidence to the contrary.

Cholera today takes advantage of breakdowns in sanitation and health infrastructure, often in the setting of natural and complex disasters. More notably, cholera has survived the transition from ancient to modern world, with the establishment of endemic foci in virtually every continent. We have learned a great deal about cholera during the past few decades. Major advances have been made in therapy, which has decreased expected case-fatality rates to <0.5%. However, we are just coming to appreciate the evolutionary capabilities of the microorganism and the complexity of transmission pathways, an understanding of which is essential to ultimate control of the disease.

## Clinical Features and Management

Clinically, cholera is a simple disease. Its manifestations result almost entirely from action of cholera toxin, a protein enterotoxin excreted by the bacterial cell. The A subunit of cholera toxin activates adenylate cyclase, causing increased Cl^–^ secretion by intestinal crypt cells and decreased NaCl-coupled absorption by villus cells and resulting in a net movement of electrolytes (and water) into the lumen of the intestine (*5*). All manifestations of the disease can be reproduced by administration of cholera toxin: in studies conducted in the 1970s at the University of Maryland (Baltimore, MD, USA), volunteers given 25 μg of pure cholera toxin had >20 L of rice-water feces; ingestion of as little as 5 μg of purified toxin resulted in 1–6 L of diarrhea in 5 of 6 volunteers (*6*).

Severity of illness varies widely. In the most severe form of the disease, cholera gravis, patients can pass >1 L of diarrheal feces per hour. Feces are passed effortlessly, with the diarrhea assuming a rice-water appearance. If volumes are not repleted, this diarrhea can result, in as little as 6–8 hours, in circulatory collapse, shock, and death. Shock, even if adequately treated, may precipitate acute renal failure. Severe acidosis results from fecal loss of bicarbonate, exacerbated by hypotension-related lactic acidosis and renal failure.

Although cholera gravis is a devastating disease, studies in the early 1970s suggested that such severe cases accounted for only 11% of total infections among persons infected with strains of the classical biotype (responsible for the sixth cholera pandemic); 59% of infections were asymptomatic or inapparent, and the remainder represented illness of mild to moderate severity. In studies during that same period, only 2% infected with seventh pandemic biotype El Tor strains had severe disease, and 75% of infected persons were asymptomatic (*7*). Although the El Tor biotype has persisted, its relative lack of virulence has not; recent studies have noted substantial increases in the percentage of patients with severe dehydration (*8*), and the percentage of asymptomatic infected patients appears to be much smaller (<50%, in a recent study by Harris et al. [9]). As described below, these observations coincide with the appearance of new atypical *V. cholerae* strains that include classical biotype genetic material within an El Tor background (*10**,**11*).

The cornerstone of therapy is replacement of lost fluid. With an infrastructure able to provide adequate rehydration therapy, case-fatality rates should be <1%, ideally <0.5%. In mild to moderate cases, rehydration can generally be successfully accomplished with oral rehydration solution. In patients who are severely dehydrated (loss of 10% of body weight) intravenous rehydration is almost always necessary; limited anecdotal reports suggest that use of intravenous therapy is becoming more frequent in areas where cholera is endemic and epidemic, consistent with concerns about increasing severity of illness. In early placebo-controlled studies, tetracycline reduced duration of diarrhea, total volume of diarrhea, and days of excretion of *V. cholerae* by >50%; more recent studies demonstrated equivalent or better results with ciprofloxacin and azithromycin. However, antimicrobial drug use is also clearly associated with development of resistance, leading to current WHO recommendations that antimicrobial agents be limited to use in patients with severe dehydration. As recently suggested by Nelson et al. (*12*), extending use of antimicrobial drugs to a larger patient group may be reasonable, particularly in light of increasing awareness of direct transmission of the microorganism from person to person, as discussed below. Zinc supplementation also has been recognized as a potentially useful adjunct to therapy; recent studies among children in Bangladesh have shown that its administration resulted in a 12% reduction in duration of diarrhea and 11% reduction in fecal volume in patients compared with controls (*13*).

## Genetics/Microbiology

*V. cholerae* is a diverse species and a natural (and common) inhabitant of estuarine environments around the world. Distribution depends on water temperature (optimal growth at water temperatures >20°C) and salinity (*14*). In contrast to most other *Vibrio* species, it is able to grow in fresh water and is often present in inland rivers and lakes in regions where it is endemic. In areas with seasonal variations in water temperatures, the microorganism shows clear seasonality: environmental counts increase during warmer periods and decline (or become nondetectable) during cold weather. In studies in Peru (*15*), *V. cholerae* counts in the Rimac River (at a site above Lima where sewage contamination was minimal) spiked ≈2 months after an initial summer rise in water temperature but then returned to a nondetectable level within 1–2 months (Technical Appendix). The reason for the sharp drop in counts after the initial spike (a pattern also seen in some ponds in Bangladesh [16]) is not clear. One hypothesis is that it is related to rapid increases in the number of *V. cholerae*–specific lytic bacteriophages in the local aquatic environment (*17**,**18*), providing natural predation as a countermeasure to the initial rapid increase in numbers of the microorganism.

*V. cholerae* can assume a variety of survival forms, including a shift to what has been termed a viable but nonculturable form, which is often associated with biofilms. Strains can also assume a rugose phenotype (identifiable on culture by a characteristic rough/wrinkled appearance), in which the microorganism produces large quantities of an amorphous exopolysaccharide, leading to formation of a biofilm that is resistant to chlorine, UV light, and other standard disinfectants (*19*). *V. cholerae* has been closely linked with copepods (binding to chitin through the action of a specific chitinase) and with zooplankton (*14*). It has also been found in association with chironomid egg masses and water hyacinth and can be carried by gulls, other birds, and mammals.

Although *V. cholerae* as a species is ubiquitous in the environment, strains responsible for the disease cholera are restricted to a fairly tight subset of strains, as reflected in clustering seen by multilocus sequence typing and sequence analysis. The key gene clusters responsible for the manifestations of cholera are associated with production of cholera toxin located within the *ctx* element (which is part of a filiamentous phage capable of movement among strains [20]) and the vibrio pathogenicity island, which includes the TCP (toxin-coregulated pilus) gene, essential for binding of the microorganism to the intestinal mucosa. Other genes common to strains with an epidemic phenotype also have been identified; however, the role of many of these genes in the pathogenesis of cholera remains to be determined (*21**,**22*). Even though virtually all strains that cause cholera produce cholera toxin and have the vibrio pathogenicity island, not all *V. cholerae* that carry 1 or both of these gene complexes cause cholera; several studies have noted the isolation of 1 or both from environmental strains that appear to lack other components of the genetic background essential for virulence in humans and epidemic spread (*22**,**23*).

The *V. cholerae* genome readily undergoes change, with extensive genetic recombination through lateral gene transfer, resulting in what have been termed shifts and drifts in the genome sequence (*21*). This genetic plasticity is reflected in the observation that feces from a single infected patient in an area where cholera is endemic almost always show evidence of infection with multiple genetically distinct *V. cholerae* strains, as defined by variable-number tandem-repeat analysis (*24*). Variability also can be seen in serotype. Traditionally, epidemic disease was thought to be confined to cholera toxin-producing strains in *V. cholerae* O group 1. However, in 1992, a new serotype, O139, was recognized as the cause of a major cholera epidemic on the Indian subcontinent (*25*); emergence was linked to replacement of the O group 1 biosynthesis cassette with a biosynthesis cassette for the O139 antigen (which also encoded material for formation of a capsule). O group 1 strains continue to predominate among epidemic isolates, but serotype clearly does not directly predict virulence; cholera-like illness (albeit without epidemic spread) is now associated with several different serotypes in addition to O1 and O139. These serotypes include O141 and O75 in the United States and O37, O10, O12, O6, and O14 in other parts of the world (*23**,**26*). Changes in serotype, in turn, appear to result from lateral transfer of the gene cassettes responsible for O-antigen biosynthesis (*23**,**26*).

Recent changes or recombinational events also have been seen in the *ctx* gene cluster, with introduction of the classical biotype *ctx* gene into an El Tor background and the appearance of strains containing multiple recombinational events that have modifications in *ctx* as well as other changes that result in loss of traditional El Tor biotype characteristics (*10**,**11**,**27*). Although nomenclature remains in flux (*11*), these new atypical strains have, at this point, entirely supplanted traditional seventh pandemic El Tor strains at a global level (including, most recently, the strain responsible for the Haiti epidemic [28]). As discussed above, these strains also appear to have major increases in virulence (potentially because of increased levels of cholera toxin production [29]), comparable with (or in excess of) clinical characteristics of the sixth pandemic classical biotype strains.

*V. cholerae* strains associated with epidemic disease can respond to changes in their immediate environment as they move from environmental reservoirs to humans and back. Of particular relevance, it has been shown that *V. cholerae* passed in human rice-water feces are in a “hyperinfectious” state (*17**,**30*); in animal studies, an infectious dose is 1–2 orders of magnitude lower than that for strains grown by using traditional in vitro methods. The hyperinfectious state lasts at least 5 hours after passage of the microorganism from patients. The physiologic basis for this effect is unclear but appears to be associated, at least in part, with changes that include down-regulation of chemotaxis genes (*31*). *V. cholerae* as it is passed from the body also up-regulates a series of genes that are not required for infection but are needed for survival in the environment. Twenty-four hours after the microorganism reaches the aquatic environment, these shifts, potentially combined with lytic phage, result in a dramatic decrease in the ability of *V. cholerae* to cause infection (*18*).

## Epidemiology/Transmission

During the 1960s, the scientific consensus was that cholera was transmitted from person to person; a great deal of attention was given to the role of convalescent and chronic carriage in transmission. During the following decades, attention shifted sharply from human carriage to environmental reservoirs, with a focus on the role of environmental factors in persistence of the disease and triggering of epidemics. However, with the advent of increasingly sensitive molecular techniques—and mathematical modeling approaches—there has been movement back toward a transmission model that recognizes the role of environmental reservoirs and direct (human-to-human) transmission. In this context, we propose the transmission model shown in the Figure.

**Figure:**
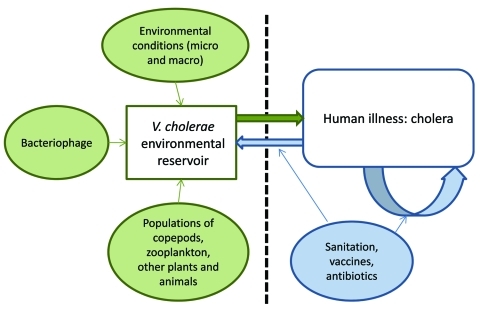
Proposed model for *Vibrio cholerae* transmission.

The aquatic environmental reservoir is critical to long-term maintenance of epidemic *V. cholerae.* These reservoirs constitute complex biological systems, with modulation of *V. cholerae* populations by environmental conditions (the local microenvironment [15,16] as well as global macroenvironmental factors, such as the El Niño/Southern Oscillation [32]); by predatory bacteriophage populations (*18*); and by fluctuations in populations of copepods and zooplankton (which can, in turn, be driven by predation by fish), binding to chironomid egg masses, water hyacinth, carriage by birds and mammals, and a host of other variables. At the same time, our data from Lima (Technical Appendix) and from Bangladesh show a significant association between a spike in numbers of *ctx*-positive *V. cholerae* in the environment and subsequent occurrence of cholera in the community (*15**,**16*), consistent with the concept of spillover of the microorganism from the environment to human populations.

On the basis of our studies in Lima and Bangladesh, peak environmental counts of *ctx*-positive *V. cholerae* from pristine areas range from 10^1^ to 10^2^ CFU/mL (*15**,**16*). The infectious dose for *V. cholerae* (classical biotype) ingested by healthy North American volunteers is in the range of 10^8^ to 10^11^ CFU/mL, which drops to 10^4^–10^8^ when the inoculum is given with bicarbonate or food (*5**,**33*). Assuming consumption of a large enough volume of contaminated material; mixing with food (with the potential for further growth in food before consumption [34,35]); and possible underlying host factors, such as mild hypochlorhydria (which can be associated with *Helicobacter pylori* infection, endemic to many developing countries) and malnutrition, transmission from environmental sources that are not heavily fecally contaminated becomes plausible. However, this infectious dose still would be at the low end of the curve, and the percentage of exposed persons becoming infected at these low levels is likely to be relatively small.

The picture changes once the microorganism is introduced into human populations. Rice-water feces contain 10^7^–10^9^ *V. cholerae* microorganisms per mL. Immediately after passage, these microorganisms are in a hyperinfectious state (further dropping the infectious dose by 1 or 2 logs), generating the opportunity for “fast” transmission of *V. cholerae* to other persons either by direct contact with feces or direct contamination of food or water within the immediate household environment. Microorganisms from feces can also reenter environmental reservoirs by fecal contamination. However, one then has to deal with dilutional effects within the environment and the striking drop in infectivity (noted above) that can occur as the microorganism adjusts to an environmental habitat (*18*). In our studies in Lima at the peak of a cholera epidemic, we found environmental counts of toxigenic *V. cholerae* of ≈10^5^/mL in areas with heavy sewage contamination, so the potential for infection from environmental sources clearly increases in settings of poor sanitation during epidemics. Nonetheless, looking at the relative counts from different sources of exposure, these observations are consistent with the hypothesis that a major transmission pathway for *V. cholerae* during an epidemic (particularly at the beginning of an epidemic) is through a “fast” pathway, taking advantage of the short-lived hyperinfectious state to move from person to person, without an intervening “slow” transmission step through the environment.

Data from a variety of sources support this hypothesis. In studies in Bangladesh that used variable-number tandem-repeat analysis (*36*), we found minimal overlap between clinical strain populations circulating in human populations and the *ctx*-positive *V. cholerae* strains that were circulating concurrently in environmental reservoirs. If the environmental reservoir was playing a major role in the ongoing epidemic, one would have expected to see the same genetic types appearing in strains from the environment and strains from patients. In mathematical models, inclusion of a fast (presumed person-to-person or person-to–household environment–to-person) transmission pathway that incorporates the short-lived hyperinfectious state results in a much better match with outbreak data than models that rely solely on a “slow” human-to–aquatic environment–to-human pathway (*37*). In subsequent work we have applied our models to data from the 2008–2009 Zimbabwe epidemic (*3*). When calculated by province, the reproductive number (R_0_) for the epidemic ranged from 1.1 to 2.7; our calculations suggest that 47%–94% of this value, dependent on province, was accounted for by fast (hyperinfectious/presumed person-to-person) transmission.

These observations underscore the need to focus prevention efforts on the short window of time after passage of feces when strains are in a hyperinfectious state. This observation translates into the need for an emphasis on households (and, in particular, an emphasis on households with index cases), where exposure to recently excreted microorganisms is most likely. This finding fits with those from earlier epidemiologic studies from Bangladesh, where risk for illness was linked with the presence of an infected person in the household, not with whether the household used clean tube well water versus potentially contaminated surface water for drinking (*38*). It is also in agreement with work by Deb et al. in Kolkata, India, which highlighted the influence of household transmission during epidemic periods and the associated need to focus on minimizing risk for contamination of water and food sources within the household (*39*).

## Summary

*V. cholerae* is a wily opponent. It can live indefinitely in aquatic reservoirs, making eradication difficult, if not impossible; readily undergoes genetic modification, permitting response to changing environmental (and human) conditions; and shifts patterns of gene expression as it moves from one local environment to another (including a shift to a hyperinfectious state immediately after passage in feces). Several mathematical models (including models developed by the Emerging Pathogens Institute, University of Florida [Gainesville, FL, USA] [3]) support the potential value of vaccines for disease control and have outlined potential strategies for their utilization (*40*). Although the environment remains a critical component of transmission, interventions focused increasingly on the household and on blocking transmission immediately after passage of feces are acutely needed. Ultimately, good sanitation (as part of a strong public health infrastructure) is the key to disease control. However, until sanitation is widespread (and the impact of natural and human-made disasters is minimized), ways in which this age-old pathogen causes disease—and ways in which it can be controlled—need to continue to be explored.

## Supplementary Material

Technical Appendix Figure depicting ctx-positive water samples and numbers of cholera cases.
