# Supplementary material for: Cholera—Modern Pandemic Disease of Ancient Lineage
Source: Emerg Infect Dis. 2011 Nov;17(11):2099–104. doi: 10.3201/eid1711.111109 (PMC3310593; doi:10.3201/eid1711.111109)
Supplement: Technical Appendix — Figure depicting ctx-positive water samples and numbers of cholera cases. [file 11-1109-Techapp_1p.pdf]

DOI: http://dx.doi.org/10.3201/eid1711.111109

Cholera—Modern Pandemic Disease of Ancient Lineage

Technical Appendix

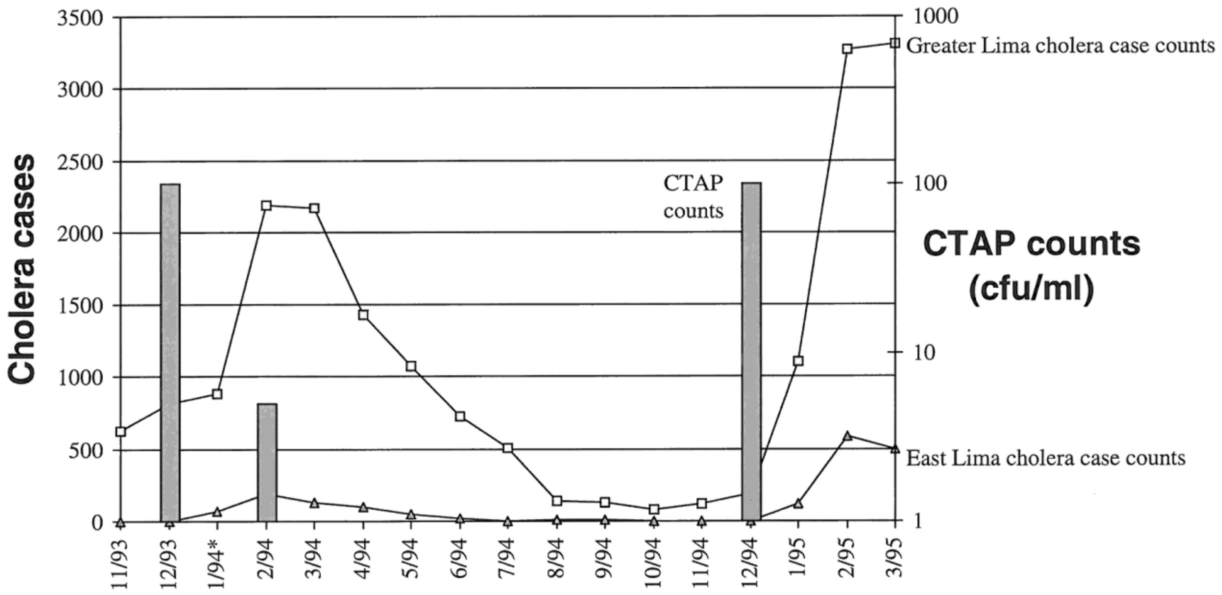

Figure. Numbers of *ctx*-positive *Vibrio cholerae* identified in water samples at a “pristine” location upriver on the Rimac River, Lima, Peru (bars, with log scale on right of figure), and numbers of cholera cases in the local community (lower line, scale on left of figure) and in greater Lima (upper line), by month. Although sampling was conducted monthly, *V. cholerae* carrying *ctx* was detected only in 12/94, 2/94, and 12/94. From Franco et al. (*23*), used with permission.
